# Non-contiguous finished genome sequence of *Bacteroides coprosuis* type strain (PC139^T^)

**DOI:** 10.4056/sigs.1784330

**Published:** 2011-04-29

**Authors:** Miriam Land, Brittany Held, Sabine Gronow, Birte Abt, Susan Lucas, Tijana Glavina Del Rio, Matt Nolan, Hope Tice, Jan-Fang Cheng, Sam Pitluck, Konstantinos Liolios, Ioanna Pagani, Natalia Ivanova, Konstantinos Mavromatis, Natalia Mikhailova, Amrita Pati, Roxane Tapia, Cliff Han, Lynne Goodwin, Amy Chen, Krishna Palaniappan, Loren Hauser, Evelyne-Marie Brambilla, Manfred Rohde, Markus Göker, John C. Detter, Tanja Woyke, James Bristow, Jonathan A. Eisen, Victor Markowitz, Philip Hugenholtz, Nikos C. Kyrpides, Hans-Peter Klenk, Alla Lapidus

**Affiliations:** 1 DOE Joint Genome Institute, Walnut Creek, California, USA; 2 Oak Ridge National Laboratory, Oak Ridge, Tennessee, USA; 3 Los Alamos National Laboratory, Bioscience Division, Los Alamos, New Mexico, USA; 4 DSMZ – German Collection of Microorganisms and Cell Cultures GmbH, Braunschweig, Germany; 5 Biological Data Management and Technology Center, Lawrence Berkeley National Laboratory, Berkeley, California, USA; 6 HZI – Helmholtz Centre for Infection Research, Braunschweig, Germany; 7 University of California Davis Genome Center, Davis, California, USA; 8 Australian Centre for Ecogenomics, School of Chemistry and Molecular Biosciences, The University of Queensland, Brisbane, Australia

**Keywords:** strictly anaerobic, non-motile, Gram-negative, mesophilic, chemoorganotrophic, *Bacteroidaceae*, GEBA

## Abstract

*Bacteroides coprosuis* Whitehead *et al.* 2005 belongs to the genus *Bacteroides*, which is a member of the family *Bacteroidaceae*. Members of the genus *Bacteroides* in general are known as beneficial protectors of animal guts against pathogenic microorganisms, and as contributors to the degradation of complex molecules such as polysaccharides. *B. coprosuis* itself was isolated from a manure storage pit of a swine facility, but has not yet been found in an animal host. The species is of interest solely because of its isolated phylogenetic location. The genome of *B. coprosuis* is already the 5^th^ sequenced type strain genome from the genus *Bacteroides*. The 2,991,798 bp long genome with its 2,461 protein-coding and 78 RNA genes and is a part of the *** G****enomic* *** E****ncyclopedia of* *** B****acteria and* *** A****rchaea * project.

## Introduction

Strain PC139^T^ (= DSM 18011 = NRRL B-41113 = JCM 13475) is the type strain of *Bacteroides coprosuis* which belongs to the large genus *Bacteroides*, which currently contains 39 members [1,2]. The species epithet is derived from the Greek noun *'kopros'* meaning 'feces' and the genitive of the Latin noun *'suis'* meaning 'of a pig'. *B. coprosuis* strain PC139^T^ was isolated from a manure storage pit of a swine facility. One other strain belonging to the same species has been isolated from the same source [2]. Many *Bacteroides* species are common inhabitants of the intestine where they help to degrade complex molecules such as polysaccharides or transform steroids [3,4]. They also play a role as beneficial protectors of the gut against pathogenic microorganisms [5]. However, so far *B. coprosuis* has not been isolated from an animal itself, therefore the exact habitat and the role the bacterium plays remains unknown. Here we present a summary classification and a set of features for *B. coprosuis* PC139^T^, together with the description of the complete genomic sequencing and annotation.

## Classification and features

A representative genomic 16S rRNA sequence of strain PC139^T^ was compared using NCBI BLAST under default settings (e.g., considering only the high-scoring segment pairs (HSPs) from the best 250 hits) with the most recent release of the Greengenes database [6] and the relative frequencies, of taxa and keywords (reduced to their stem [7]) were determined, weighted by BLAST scores. The most frequently occurring genus was *Bacteroides* (100.0%) (20 hits in total). Regarding the single hit to sequences from members of the species, the identity within HSPs was 99.9%, whereas the coverage by HSPs was 98.0%. Regarding the twelve hits to sequences from other members of the genus, the average identity within HSPs was 92.9%, whereas the average coverage by HSPs was 62.1%. Among all other species, the one yielding the highest score was *B. propionicifaciens*, which corresponded to an identity of 94.6% and an HSP coverage of 84.5%. The highest-scoring environmental sequence was AF445205 ('Swine fecal isolate str. FPC111'), which showed an identity of 99.8% and an HSP coverage of 100.0%. The most frequently occurring keywords within the labels of environmental samples which yielded hits were 'human' (6.3%), 'fecal' (5.5%), 'effect' (4.4%), 'antibiot, deep, gut, microbiota, pervas, sequenc' (4.3%) and 'feedlot' (4.2%) (230 hits in total). Environmental samples which yielded hits of a higher score than the highest scoring species were not found.

**Figure 1:**
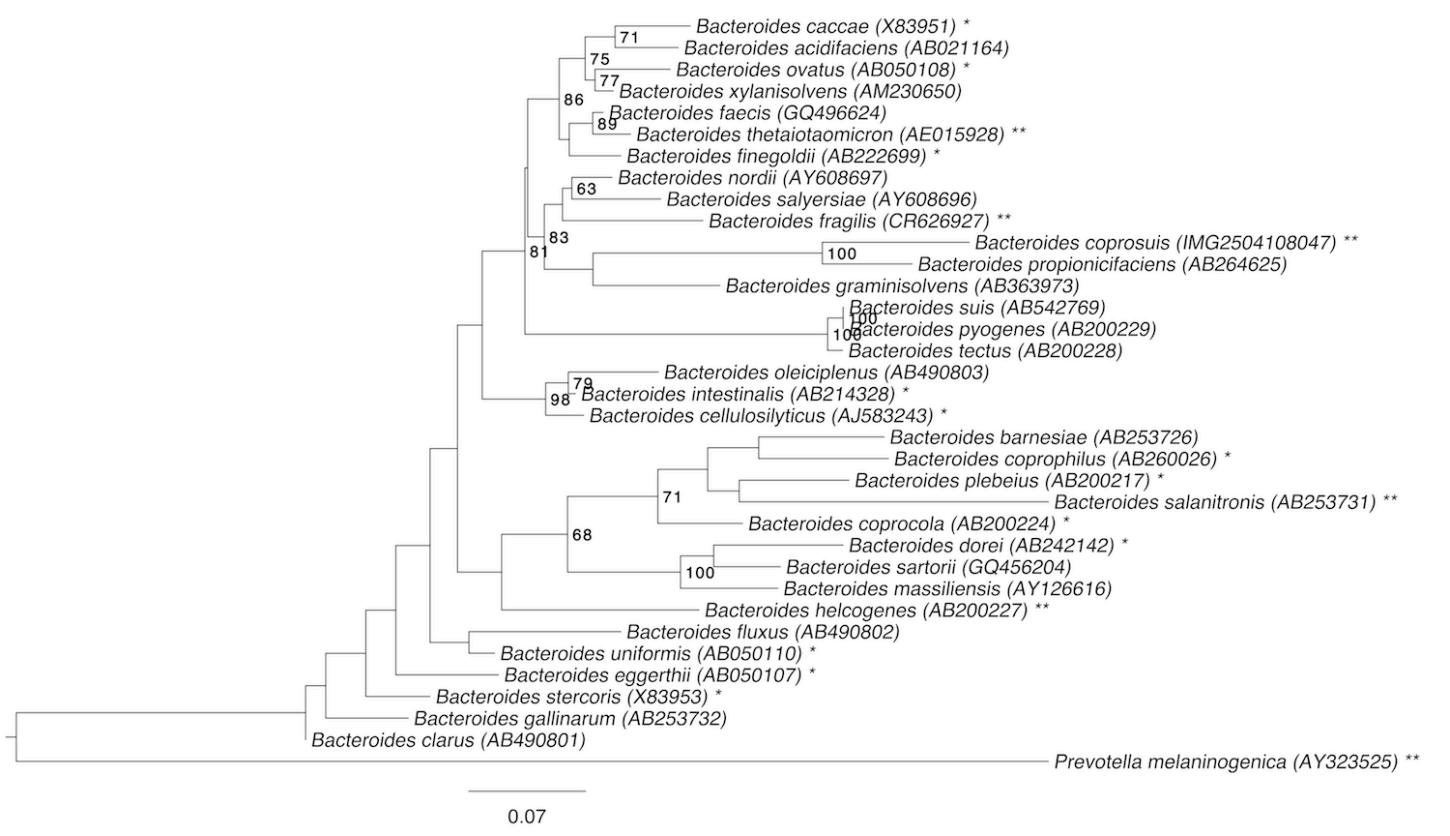
Phylogenetic tree highlighting the position of *B. coprosuis* relative to the other type strains within the genus *Bacteroides*. The tree was inferred from 1,412 aligned characters [8,9] of the 16S rRNA gene sequence under the maximum likelihood criterion [10] and rooted in accordance with the current taxonomy. The branches are scaled in terms of the expected number of substitutions per site. Numbers to the right of bifurcations are support values from 1,000 bootstrap replicates [11] if larger than 60%. Lineages with type strain genome sequencing projects registered in GOLD [12] and unpublished are marked with one asterisk, those listed as published (as well as the target genome) with two asterisks [13-17], and CP002122/3 for *Prevotella melaninogenica*.

Figure 1 shows the phylogenetic neighborhood of *B. coprosuis* in a 16S rRNA based tree. The sequences of the three 16S rRNA gene copies in the genome differ from each other by up to seven nucleotides, and differ by up to six nucleotides from the previously published 16S rRNA sequence (AF319778).

**Figure 2:**
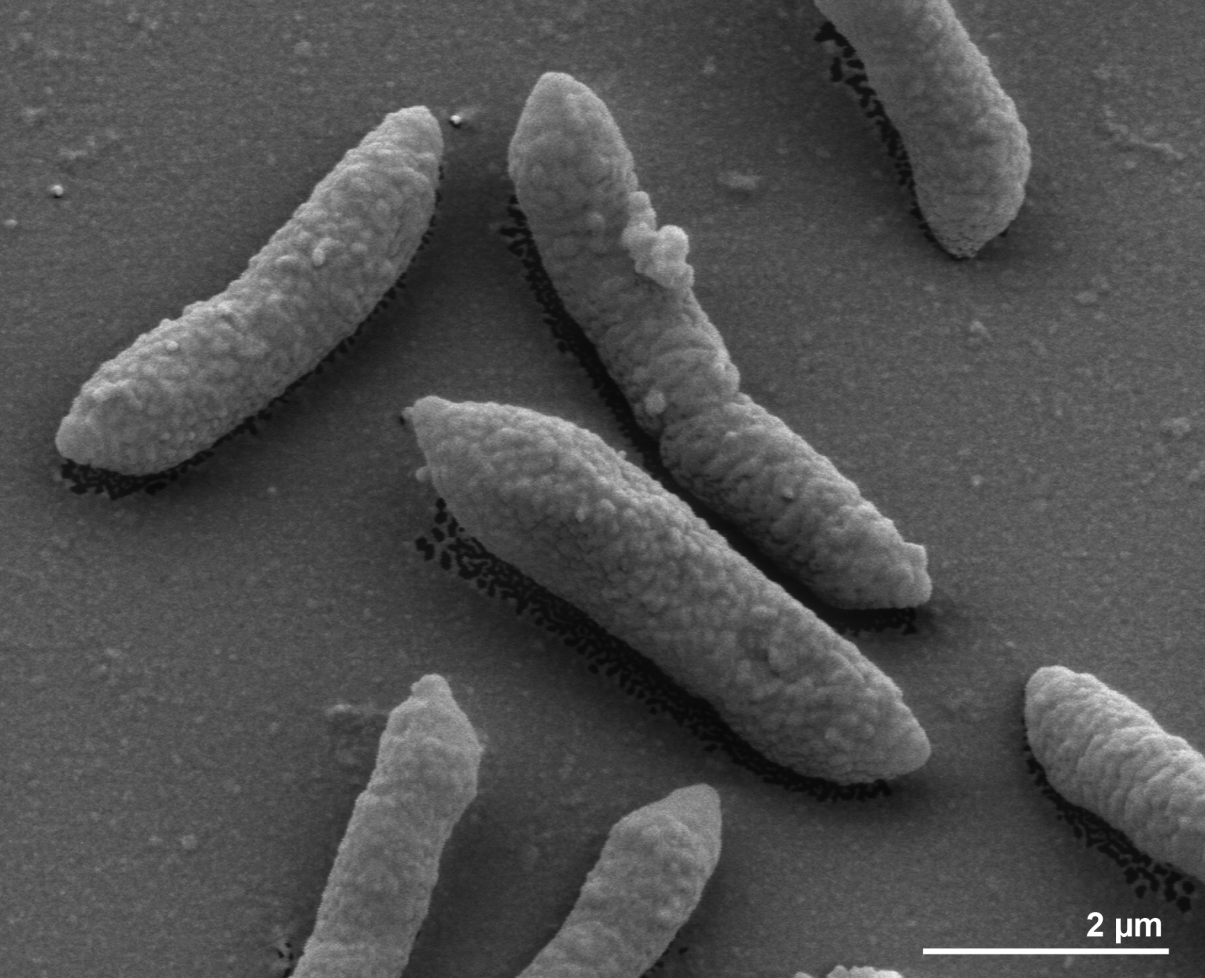
Scanning electron micrograph of *B. coprosuis* PC139^T^

The cells of *B. coprosuis* are generally rod-shaped (0.5-1.5 × 0.8-3.0 µm) with tapered ends (Figure 2). The cells are usually arranged singly or in pairs [2]. *B. coprosuis* is a Gram-negative and non spore-forming bacterium (Table 1). The organism is finally described to be non-motile; only four genes associated with motility have been found in the genome (see below). The organism grows at temperatures from 25 to 37°C, but not at 42°C or higher; the optimal temperature is 37°C [2]. *B. coprosuis* is a strictly anaerobic chemoorganotroph and is able to grow on media containing glucose, maltose and chondroitin sulfate but, no growth was observed on arabinogalactan, arabinose, cellobiose, corn-fibre xylan, corn-spelt xylan, fructose or xylose [2]. The organism produces acid from mannose, but not from raffinose. It hydrolyzes esculin and starch, but does not liquify gelatin, reduce nitrate nor produce indole from tryptophan [2]. Growth is possible in the presence of 20% bile [2]. Major fermentation products from glucose are acetic acid (8.0-15.0 mM), succinic acid (7.5-10.0 mM) and propionic acid (4.0-22.0 mM) [2]. *B. coprosuis* shows activity for alkaline and acid phosphatase, α-fucosidase, β-galactosidases, α- and β-glucosidases, *N*-acetyl-β-glucosaminidase, chymotrypsin, esterase C4, ester lipase C8, lipase C14, cystine arylamidase, leucyl glycine arylamidase, alanine arylamidase, arginine arylamidase and glutamyl glutamic acid arylamidase. No activity was detected for urease, catalase, oxidase, trypsin, arginine dihydrolase, β-galactosidase 6-phosphate, β-glucuronidase, α-arabinosidase, α-mannosidase and glutamic acid, glycine, histidine, leucine, phenylalanine, proline, pyroglutamic acid, serine, tyrosine and valine arylamidase [2]. *B. coprosuis* is resistant to ampicillin (100 µg/ml), cefoxitin (20 µg/ml), erythromycin (10 µg/ml), gentamicin (200 µg/ml) and tetracycline (3 µg/ml).

**Table 1:** Classification and general features of *B. coprosuis* PC139^T^ according to the MIGS recommendations [18].

MIGS ID	Property	Term	Evidence code
	Current classification	Domain *Bacteria*	TAS [19]
		Phylum *Bacteroidetes*	TAS [20]
		Class *'Bacteroidia'*	TAS [21]
		Order *'Bacteroidales'*	TAS [22]
		Family *Bacteroidaceae*	TAS [23,24]
		Genus *Bacteroides*	TAS [23,25-28]
		Species *Bacteroides coprosuis*	TAS [2]
		Type strain PC139	TAS [2]
	Gram stain	negative	TAS [2]
	Cell shape	rod-shaped	TAS [2]
	Motility	non-motile	TAS [2]
	Sporulation	none	TAS [2]
	Temperature range	25°C–37°C	TAS [2]
	Optimum temperature	37°C	TAS [2]
	Salinity	not reported	
MIGS-22	Oxygen requirement	strictly anaerobic	TAS [2]
	Carbon source	carbohydrates	TAS [2]
	Energy metabolism	chemoorganotroph	TAS [2]
MIGS-6	Habitat	most probably *Sus scrofa domestica*	TAS [2]
MIGS-15	Biotic relationship	free-living	NAS
MIGS-14	Pathogenicity	none	NAS
	Biosafety level	1	TAS [29]
	Isolation	pig feces, manure storage pit	TAS [2]
MIGS-4	Geographic location	USA	TAS [2]
MIGS-5	Sample collection time	2005 or before	NAS
MIGS-4.1	Latitude	not reported	
MIGS-4.2	Longitude	not reported	
MIGS-4.3	Depth	not reported	
MIGS-4.4	Altitude	not reported	

Evidence codes - IDA: Inferred from Direct Assay (first time in publication); TAS: Traceable Author Statement (i.e., a direct report exists in the literature); NAS: Non-traceable Author Statement (i.e., not directly observed for the living, isolated sample, but based on a generally accepted property for the species, or anecdotal evidence). These evidence codes are from of the Gene Ontology project [30]. If the evidence code is IDA, then the property was directly observed by one of the authors or an expert mentioned in the acknowledgements.

### Chemotaxonomy

Little chemotaxonomic information is available for strain PC139^T^. Thus far, only the fatty acid composition has been elucidated. The major fatty acids found were *anteiso-*C_15:0_ (31%), *iso-*C_17:0 _3-OH (17%), *iso-*C_17:0_ (10%), *iso*-C_15:0_ (8%) and C_15:0_ (8%). Fatty acids C_16:0_ (3.5%), *anteiso-*C_17:0_ (3%), C_18:1_ω9c (2%), C_17:0_ (2%), *anteiso*-C_17:1_ω9c (2%), C_18:0_ (1%) and *iso-*C_13:0_ (1%) were found in minor amounts [2].

## Genome sequencing and annotation

### Genome project history

This organism was selected for sequencing on the basis of its phylogenetic position [31], and is part of the *** G****enomic* *** E****ncyclopedia of* *** B****acteria and* *** A****rchaea * project [32]. The genome project is deposited in the Genomes On Line Database [12] and the complete genome sequence is deposited in GenBank. Sequencing, finishing and annotation were performed by the DOE Joint Genome Institute (JGI). A summary of the project information is shown in Table 2.

**Table 2:** Genome sequencing project information

**MIGS ID**	**Property**	**Term**
MIGS-31	Finishing quality	Non-contiguous finished
MIGS-28	Libraries used	Three genomic libraries: one 454 pyrosequence standard library, one 454 PE library (7 kb insert size), one Illumina library
MIGS-29	Sequencing platforms	Illumina GAii, 454 GS FLX Titanium
MIGS-31.2	Sequencing coverage	283.0 × Illumina; 36.6 × pyrosequence
MIGS-30	Assemblers	Newbler version 2.3-PreRelease-09-14-2009, Velvet version 0.7.63, phrap version 4.24
MIGS-32	Gene calling method	Prodigal 1.4, GenePRIMP
	INSDC ID	AFFW00000000
	Genbank Date of Release	May 12, 2011
	GOLD ID	Gi03975
	NCBI project ID	40779
	Database: IMG-GEBA	2503982039
MIGS-13	Source material identifier	DSM 18011
	Project relevance	Tree of Life, GEBA

### Growth conditions and DNA isolation

*B. coprosuis* PC139^T^, DSM 18011, was grown anaerobically in DSMZ medium 104 (modified PYG-medium) + rumen fluid (200µl/10 ml) [33] at 37°C. DNA was isolated from 0.5-1 g of cell paste using MasterPure Gram-positive DNA purification kit (Epicentre MGP04100) following the standard protocol as recommended by the manufacturer with modification st/DL for cell lysis as described in Wu *et al*. 2009 [32]. DNA is available through the DNA Bank Network [34].

### Genome sequencing and assembly

The genome was sequenced using a combination of Illumina and 454 sequencing platforms. All general aspects of library construction and sequencing can be found at the JGI website [35]. Pyrosequencing reads were assembled using the Newbler assembler version 2.3-PreRelease-09-14-2009 (Roche). The initial Newbler assembly consisting of 100 contigs in two scaffolds was converted into a phrap assembly [36] by making fake reads from the consensus, to collect the read pairs in the 454 paired end library. Illumina GAii sequencing data (920.8 Mb) was assembled with Velvet, version 0.7.63 [37] and the consensus sequences were shredded into 1.5 kb overlapped fake reads and assembled together with the 454 data. The 454 draft assembly was based on 109.0 Mb 454 draft data and all of the 454 paired end data. Newbler parameters are -consed -a 50 -l 350 -g -m -ml 20. The Phred/Phrap/Consed software package [36] was used for sequence assembly and quality assessment in the subsequent finishing process. After the shotgun stage, reads were assembled with parallel phrap (High Performance Software, LLC). Possible mis-assemblies were corrected with gapResolution [35], Dupfinisher, or sequencing cloned bridging PCR fragments with subcloning or transposon bombing (Epicentre Biotechnologies, Madison, WI) [38]. Gaps between contigs were closed by editing in Consed, by PCR and by Bubble PCR primer walks (J.-F.Chang, unpublished). A total of 193 additional reactions and four shatter libraries were necessary to close gaps and to raise the quality of the finished sequence. Illumina reads were also used to correct potential base errors and increase consensus quality using a software Polisher developed at JGI [39]. The error rate of the completed genome sequence is less than 1 in 100,000. Together, the combination of the Illumina and 454 sequencing platforms provided 319.6 × coverage of the genome. The final assembly contained 252,927 pyrosequence and 24,365,026 Illumina reads.

### Genome annotation

Genes were identified using Prodigal [40] as part of the Oak Ridge National Laboratory genome annotation pipeline, followed by a round of manual curation using the JGI GenePRIMP pipeline [41]. The predicted CDSs were translated and used to search the National Center for Biotechnology Information (NCBI) nonredundant database, UniProt, TIGR-Fam, Pfam, PRIAM, KEGG, COG, and InterPro databases. Additional gene prediction analysis and functional annotation was performed within the Integrated Microbial Genomes - Expert Review (IMG-ER) platform [42].

## Genome properties

The genome consists of a 2,991,798 bp long circular chromosome (in one contig with one remaining unclosed sequencing gap), with a G+C content of 35.0% (Table 3). Of the 2,539 genes predicted, 2,461 were protein-coding genes, and 78 RNAs; 68 pseudogenes were also identified. The majority of the protein-coding genes (66.4%) were assigned with a putative function while the remaining ones were annotated as hypothetical proteins. The distribution of genes into COGs functional categories is presented in Table 4.

**Table 3:** Genome Statistics

**Attribute**	Value	% of Total
Genome size (bp)	2,991,798	100.00%
DNA coding region (bp)	2,551,700	85.29%
DNA G+C content (bp)	1,046,824	34.99%
Number of replicons	1	
Extrachromosomal elements	0	
Total genes	2,539	100.00%
RNA genes	78	3.07%
rRNA operons	3	
Protein-coding genes	2,461	96.93%
Pseudo genes	68	2.68%
Genes with function prediction	1,686	66.40%
Genes in paralog clusters	272	10.71%
Genes assigned to COGs	1,631	64.24%
Genes assigned Pfam domains	1,795	70.70%
Genes with signal peptides	669	26.35%
Genes with transmembrane helices	580	22.84%
CRISPR repeats	1	

**Table 4:** Number of genes associated with the general COG functional categories

**Code**	**value**	**%age**	**Description**
J	143	8.1	Translation, ribosomal structure and biogenesis
A	0	0.0	RNA processing and modification
K	84	4.8	Transcription
L	140	8.0	Replication, recombination and repair
B	0	0.0	Chromatin structure and dynamics
D	18	1.0	Cell cycle control, cell division, chromosome partitioning
Y	0	0.0	Nuclear structure
V	34	1.9	Defense mechanisms
T	59	3.4	Signal transduction mechanisms
M	154	8.8	Cell wall/membrane/envelope biogenesis
N	4	0.2	Cell motility
Z	0	0.0	Cytoskeleton
W	0	0.0	Extracellular structures
U	41	2.3	Intracellular trafficking, secretion, and vesicular transport
O	55	3.1	Posttranslational modification, protein turnover, chaperones
C	113	6.4	Energy production and conversion
G	119	6.8	Carbohydrate transport and metabolism
E	140	8.0	Amino acid transport and metabolism
F	64	3.7	Nucleotide transport and metabolism
H	111	6.3	Coenzyme transport and metabolism
I	44	2.5	Lipid transport and metabolism
P	118	6.7	Inorganic ion transport and metabolism
Q	14	0.8	Secondary metabolites biosynthesis, transport and catabolism
R	197	11.2	General function prediction only
S	102	5.8	Function unknown
-	908	35.7	Not in COGs

## Insights from the genome sequence

**Figure 3:**
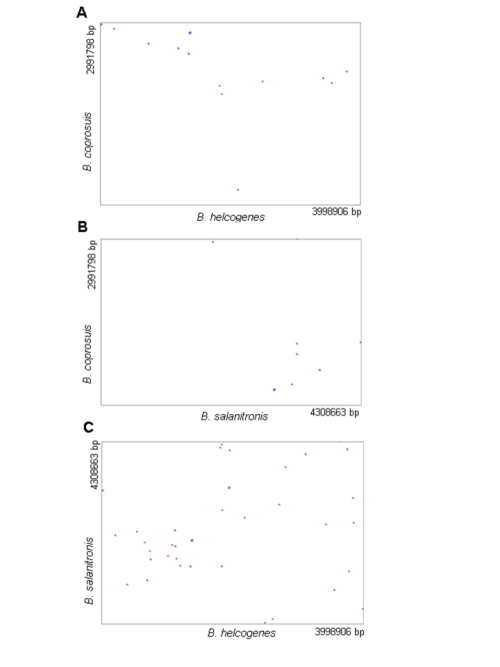
Synteny dot plots based on the genome sequences of A: *B*. *coprosuis* and *B. helcogenes*; B: *B. coprosuis* and *B. salanitronis*; C: *B. salanitronis* and *B. helcogenes*. Blue dots represent regions of similarity found on parallel strands and red dots show regions of similarity found on antiparallel strands.

Figure 3 shows synteny dot plots of three *Bacteroides* type strain genomes (*B*. *helcogenes*, *B. salanitronis*, *B. coprosuis*) with each other. *In* all three pairwise comparisons it becomes visible that there does not exist a high collinearity between these species of the genus *Bacteroides*.

The Genome-to-Genome Distance Calculator, GGDC [43,44] was used for the estimation of the overall similarity between the three *Bacteroides* genomes. The system calculates the distances by comparing the genomes to obtain HSPs (high-scoring segment pairs) and interfering distances from the set of formulas (1 HSP length / total length; 2 identities / HSP length; 3 identities / total length). The comparison of *B. coprosuis* with *B. helcogenes* and *B. salanitronis* revealed that only 6.1% and 3.3%, respectively, of the average of the genome lengths are covered with HSPs. The identity within the HSPs was 82.3% and 82.1%, respectively, whereas the identity over the whole genome was 5.0% and 2.7%, respectively. The comparison of *B. salanitronis* with *B. helcogenes* revealed that 11.4% of the genome is covered with HSPs, with an identity within in the HSPs of 81.4% and an identity over the whole genome of 9.2%. According to these calculations the similarity between *B*. *salanitronis* and *B*. *helcogenes* is higher than the similarity between *B. coprosuis* and *B. salanitronis* as well as the similarity between *B. coprosuis* and *B*. *helcogenes.*

**Figure 4:**
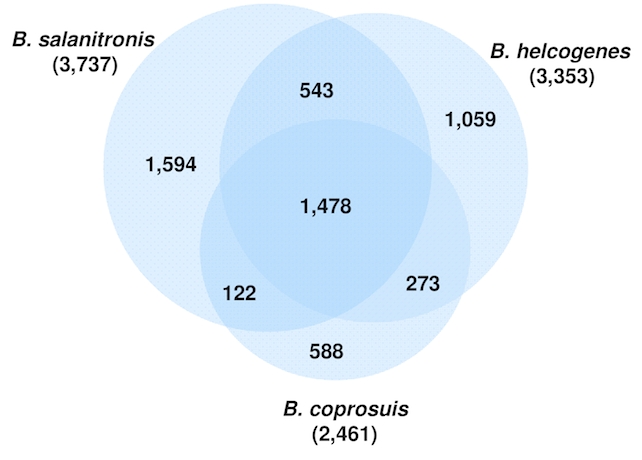
Venn diagram depicting the intersections of proteins sets (total numbers in parentheses) of the three sequenced *Bacteroides* genomes.

The genome size of *B*. *coprosuis* (3 Mb) is significantly smaller than those of *B*. *helcogenes* (4 Mb) and *B. salanitronis* (4.3 Mb) and the G+C-content of the *B*. *coprosuis* genome (35%) is much lower than the G+C-content of *B*. *helcogenes* (45%) and *B. salanitronis* (46%) genomes. The Venn-diagram (Figure 4) shows the number of shared genes. *B. salanitronis* and *B*. *helcogenes* share a great number of genes (543 genes) that are not present in *B. coprosuis*. This fraction of genes includes genes coding for glycoside hydrolases, which are responsible for the degradation of polysaccharides. Only 12 glycoside hydrolases were identified in the genome of *B. coprosuis*, whereas the number of glycoside hydrolases identified in *B. helcogenes* and *B. salanitronis*, is much higher, 38 and 45, respectively. Whereas only two transposase genes were identified in the genome of *B*. *helcogenes*, the genomes of *B. coprosuis* and *B. salanitronis* encode a high number of transposases, 34 and 29, respectively. As a consequence, genome rearrangements can occur, which result in a quite dynamic genome structure (Figure 4). Only a small number of the genes (588 genes) found in *B. coprosuis* are not present in *B*. *helcogenes* and/or *B. salanitronis*.
